# Discovery and validation of circulating miRNAs for the clinical prognosis of severe dengue

**DOI:** 10.1371/journal.pntd.0010836

**Published:** 2022-10-17

**Authors:** Umaporn Limothai, Nattawat Jantarangsi, Natthasit Suphavejkornkij, Sasipha Tachaboon, Janejira Dinhuzen, Watchadaporn Chaisuriyong, Supachoke Trongkamolchai, Mananya Wanpaisitkul, Chatchai Chulapornsiri, Anongrat Tiawilai, Thawat Tiawilai, Terapong Tantawichien, Usa Thisyakorn, Nattachai Srisawat

**Affiliations:** 1 Excellence Center for Critical Care Nephrology, King Chulalongkorn Memorial Hospital, Bangkok, Thailand; 2 Center of Excellence in Critical Care Nephrology, Faculty of Medicine, Chulalongkorn University, Bangkok, Thailand; 3 Tropical Medicine Cluster, Chulalongkorn University, Bangkok, Thailand; 4 Department of internal medicine, Buddhachinaraj hospital, Phitsanulok, Thailand; 5 Department of internal medicine, Uttaradit hospital, Uttaradit, Thailand; 6 Banpong Hospital, Ratchaburi, Thailand; 7 Photharam Hospital, Ratchaburi, Thailand; 8 Division of Infectious Diseases, Department of Medicine, Faculty of Medicine, Chulalongkorn University, Bangkok, Thailand; 9 Division of Nephrology, Department of Medicine, Faculty of Medicine, King Chulalongkorn Memorial Hospital, Bangkok, Thailand; 10 Center for Critical Care Nephrology, The CRISMA Center, Department of Critical Care Medicine, University of Pittsburgh, School of Medicine, Pittsburgh, Pennsylvania, United States of America; 11 Academy of Science, Royal Society of Thailand, Bangkok, Thailand; University of Peradeniya Faculty of Medicine, SRI LANKA

## Abstract

**Background:**

Early prognostic markers of severe dengue may improve case management and reduce dengue-related mortalities. This study aimed to identify circulating microRNAs (miRNAs) as biomarkers for predicting severe dengue.

**Methodology:**

Serum samples from dengue-infected patients were collected on the first day of admission. Patients were followed up for 14 days after admission to determine the final diagnosis. Participants were divided into non-severe and severe dengue, as defined by WHO 2009 criteria. Circulating microtranscriptome analysis was performed using NanoString miRNA Expression Assay. The expression level of candidate miRNAs were then validated by quantitative reverse transcription-PCR method.

**Principal findings:**

The discovery cohort (N = 19) lead to the identification of 37 differentially expressed miRNAs between the two groups. Six up-regulated candidate miRNAs were selected and further validated in the larger cohort (N = 135). MiR574-5p and miR1246 displayed the highest diagnostic performance in discriminating between severe from non-severe dengue (ROC-AUC = 0.83). Additionally, miR574-5p and miR1246 had high sensitivity and high negative predictive value for detecting severe dengue. Multivariate analysis suggested that serum miR574-5p was an independent predictor of severe dengue (odds ratio 3.30, 95% CI 1.81–6.04; *p*<0.001).

**Conclusion:**

Our study indicated that circulating miRNAs, especially miR-574-5p and miR-1246, might be a promising diagnostic and prognostic biomarker for severe dengue upon hospital admission, especially when using these biomarkers on days 1 to 2 before the onset of severe dengue complications.

## Author summary

Dengue infection, a mosquito-borne disease, is an expanding global problem. It has a broad clinical spectrum that includes severe and non-severe clinical manifestations with a high risk of death. Identifying early prognostic markers of severe complications may improve case management and reduce dengue-related mortalities. The circulating microRNA (miRNA) profile has been widely used to identify potential biomarkers against viral infections. Our data revealed that the circulating miRNA expression pattern of severe dengue patients was significantly different from the non-severe group. In addition, circulating miRNAs, especially miR-574-5p and miR-1246, could be promising diagnostic and prognostic biomarkers for severe dengue. These data have implications for developing biomarkers for clinical use and could improve risk prediction in dengue patients.

## Introduction

Dengue infection, a mosquito-borne disease, is an expanding global problem that annually affected approximately 100 million people [1]. The disease is caused by four closely related dengue virus (DENV) serotypes known as DENV-1 to DENV-4 [1]. Dengue is recognized as a disease entity with different clinical presentations and unpredictable clinical outcomes [2]. It has a broad clinical spectrum that includes severe and non-severe clinical manifestations with a high risk of death [3,4]. Most DENV-infected patients can recover following a self-limited, non-severe clinical course; however, a small proportion may progress to severe disease [2]. Early predictive biomarkers of severe complications may improve case management, reduce unnecessary hospital admissions, and lower dengue-related mortality [2]. Numerous studies have been conducted to identify potential severity biomarkers [5–7]. A combination of gene expression markers has been proposed to detect severe dengue [8,9]. Currently, no single reliable biomarker of severe dengue exists in clinical practice.

MicroRNAs (miRNAs) are small non-coding RNAs spanning 20–22 nucleotides that play a significant role in the posttranscriptional regulation of gene expression [10]. Each miRNA can bind to diverse-sequence mRNA with varying degrees of complementarity, leading to the ability to control hundreds of genes simultaneously upon environmental changes [11]. This type of fine-tune adaptation and response results in specific profiles of the transcriptome, both for genes and miRNAs. Defined expression patterns can be used as biomarkers, especially in disease diagnosis [12,13]. Previous studies reported differential expression of miRNAs in dengue patients and infected cultured cells [14–17]. However, the role of miRNAs in severe dengue is still not completely understood.

The present study aimed to compare miRNAs expression profiles using the NanoString platform in serum samples of patients with non-severe and severe dengue under the current WHO dengue classification methods and then validate selected candidate miRNAs in a large-scale cohort by RT-qPCR.

## Methods

### Ethics statement

This research study has been approved by the Institutional Review Board, Faculty of Medicine, Chulalongkorn University (IRB No. 458/62), and the Ethics Committee of Banpong Hospital (REC No. 009/2562) and Potharam Hospital (REC No. 32/2562). Written informed consent was obtained from all subjects ≥18 years old and parents of subjects <18 years old. The study was conducted according to the Helsinki Declaration and Good Clinical Practice guidelines.

### Patients and study design

This study is a multi-center prospective observational study conducted at two hospitals in Ratchaburi province and one in Bangkok, Thailand, between 1^st^ September 2019 to 31^st^ December 2020. Suspected DENV-infected patients were screened for the study with the following criteria: admitted to the participant hospital and consented to participate (parental consent was obtained for patients of age ≤18 years). Blood samples were collected from the suspected dengue patients on the first day of enrollment (9 mL from patients of age ≤15 years and 18 mL from patients of age >15 years).

For serum collection, whole blood was drawn into a 10 mL serum collection tube (red topped tube) and sat in an undisturbed upright position for at least 15–30 minutes at room temperature to allow the blood to clot. Then, the tube was centrifuged for 10 minutes at 3,000 RPM. The liquid component (serum) was immediately transferred into a clean polypropylene tube. For plasma collection, whole blood was drawn into a 10 mL heparinized tube (green topped tube) and inverted 6 to 8 times. Plasma was separated by centrifugation at 3,000 RPM for 10 minutes. After centrifugation, the liquid component (plasma) was immediately transferred into a clean polypropylene tube [18]. Serum and plasma samples were stored in aliquots at -80°C until further analysis.

Serum sample on the first day of enrollment was used for NS1, IgG/IgM screening, detection of genomic dengue RNA and confirmation of its serotype, and quantification of miRNAs. Among the screened patients, only laboratory-confirmed dengue patients [2] were included in the study. Patients were excluded if they had other infectious diseases.

The sample size was calculated based on the ROC curve [19,20] using MedCalc (MedCalc Software Ltd., Ostend, Belgium) [21]. The following values were used for the calculation: type-1 error = 0.05; type-2 error = 0.20 (power is 80%); target area under the curve (AUC) = 0.7; AUC null hypothesis = 0.5; ratio of negative/positive cases = 660/138 (non-severe /severe dengue) [22]. The minimal total sample size derived was 116 cases, including 96 non-severe cases and 20 severe cases. However, we decided to enroll 135 patients to allow for potential dropouts.

In this study, we also included healthy controls (N = 21) and other febrile illness (OFI) patients (N = 23) as additional control groups.

### Classification criteria

Patients were classified as having dengue based on the presence of IgM antibodies (Ab), nonstructural protein 1 (NS1), or detection of genomic dengue RNA using conventional reverse transcription-polymerase chain reaction (RT-PCR). Samples were screened using a one-step immunochromatographic assay designed to detect both dengue virus NS1 antigen and antibodies (IgG/IgM) to DENV (SD BIOLINE Dengue Duo kit, catalog number 11FK46, SD Bioline, Korea) according to the manufacturer’s instructions.

Viral RNA was also extracted from serum specimens using QIAamp Viral RNA Mini Kit (QIAGEN, Germany) according to the manufacturer’s instructions for the molecular detection of DENV and confirmation of its serotype as previously described [23–25]. Briefly, Target viral RNA was converted to complementary DNA (cDNA) prior to enzymatic DNA amplification by reverse transcriptase (RT) and the dengue virus downstream consensus primer, homologous to the genomic RNA of the four serotypes. Subsequent *Taq* polymerase amplification was performed on the resulting cDNA with the upstream dengue virus consensus primer. The amplification reaction was performed by combining the reverse transcription of viral RNA and the subsequent *Taq* polymerase amplification in a single reaction vessel. A specimen containing DENV was identified by the detection of a DNA band of 511 base pairs (bp). The serotype was then determined by Nested PCR using specific primer sets to amplify serotype-specific fragments from the regions encoding the capsid of DENV. A specimen containing DENV-1, 2, 3 or 4 was identified by the detection of a DNA band of 482, 119, 290, or 392 bp, respectively.

All confirmed dengue cases were further categorized as non-severe without WS (dengue infection or DI), non-severe dengue with a warning sign (DWS), and severe dengue (SD) according to 2009 WHO criteria [2] using clinical and biological data recorded throughout the entire hospitalization period. The DWS was defined as the presence of abdominal pain or tenderness, persistent vomiting, clinical fluid accumulation, mucosal bleed, lethargy or restlessness, liver enlargement, and laboratory finding of increasing hematocrit concurrent with a rapid decrease in platelet count [2]. The SD was defined by at least one of the following: (i) severe plasma leakage leading to shock (narrow pulse pressure (pulse pressure ≤ 20 mmHg), hypotension (systolic blood pressure < 90 mmHg), or elevated hematocrit from baseline ≥20% plus tachycardia (heart rate >100 bpm)), (ii) severe bleeding (as evaluated by clinicians), (iii) severe organ impairment (liver: aspartate transaminase (AST) or alanine aminotransferase (ALT) ≥1,000 U/L, central nervous system (CNS): impaired consciousness, heart, kidney, and other organs).

Healthy donor serum samples obtained from apparently healthy volunteers with no history of any infection or illness were provided by the National Blood Center, Thai Red Cross Society, Bangkok, Thailand. All volunteers were confirmed to be healthy by physical examination and negative serology testing for HIV, HBV, HCV, dengue NS1, IgM, and IgG. The OFI patients were defined as those who came to hospitals with fever but were serologically negative for dengue NS1, IgM, and IgG.

### RNA isolation from serum samples

Total RNA was extracted from 200 μL of serum sample using miRNeasy Serum/Plasma Kit (Qiagen, Gaithersburg, MD, USA) according to the manufacturer’s protocol. The RNA concentration and purity were evaluated using the NanoDrop 2000 spectrophotometer (Thermo Scientific, USA).

### Microtranscriptome analysis

A total of 19 serum samples were randomly selected to investigate the expression profile of 798 human miRNAs using the nCounter1Human v3 miRNA Expression Assays (NanoString Technologies, Seattle, USA). The patients were divided into two groups based on their final diagnosis, including non-severe (N = 11) and severe dengue groups (N = 8). The non-severe group was classified into two subgroups including DI (n = 5) and DWS (n = 6). Approximately 100 ng of total RNA was preprocessed according to the manufacturer’s protocol. Raw count data were collected and captured by the nCounter Digital Analyzer for image capture (280 fields of view). The miRNA data analysis was performed using nSolver Analysis (version 4.0) software. Each miRNA count data was subtracted from the geometric mean of the negative controls. Profiling data were then normalized by the geometric mean of the positive controls and the geometric mean of the top 100 most highly-expressed microRNAs. Differential miRNA expression between groups were defined by absolute log_2_ fold-change ≥1.5 and *p*-values <0.05. The raw datasets of this assay are deposited at the NCBI Gene Expression Omnibus (GEO) under accession number GSE190749.

### Validation of candidate miRNA by RT-qPCR

Top candidate miRNAs obtained from the micro-transcriptome profiles were selected for RT-qPCR validation in an additional set of serum samples (N = 135, including 112 and 23 samples from the non-severe and severe groups, respectively). The non-severe group was classified into two subgroups including DI (n = 69) and DWS (n = 43). The selection of candidate miRNAs was based on the consistency of up-regulated expressions (log2 fold-change ≥1.5, *p*-values <0.05) in serum samples of severe dengue patients. Following these criteria, six upregulated miRNAs (miR-122-5p, miR-574-5p, miR-424-5p, miR-1303, miR-30d-5p and miR-1246) were selected for further RT-qPCR validation. Total RNA was polyadenylated with synthesis stem-loop-poly A. After polyadenylation, reverse transcription to cDNA was performed using RevertAid First Strand cDNA Synthesis Kit (Cat No. 1622, Thermo Scientific, USA). The miRNA levels were quantified from cDNA in duplicate using SYBR Green (Luna Universal qPCR Master Mix, Cat No. M3003, New England Biolabs, Inc., USA) and real-time PCR (StepOnePlus Real-time PCR System, Applied Biosystems, USA) as previously described [26]. Primer sequences for miRNAs used in the study were shown in S1 Table and RT-qPCR standardization were shown in S1 Fig. To identify suitable reference genes. Firstly, we performed a literature review on reference genes used for data normalization of human circulating miRNA. Based on several studies, miR-16-5p represented one of the most stably expressed miRNAs in serum samples [27,28]. Secondly, we checked our Nanostring data and found miR-16 was detectable in all samples and not differently expressed between severe and non-severe dengue groups (*p*-value of <0.05). Finally, we performed a pilot study on this miRNA to determine the study groups’ variation. The result showed no variation among the study groups, as shown in S2 Fig. Finally, the relative miRNA expression level was calculated by the 2^-ΔΔCT^ method.

### Statistical analysis

The NanoString analysis was performed using nSolver Analysis Software (Version 4.0). Continuous variables are presented as mean ± standard deviation in the case of normal distribution and as a median and interquartile range for non-normally distributed variables. The continuous data were analyzed using the Student’s t-test or one-way ANOVA for parametric valuables and the Mann–Whitney U test or Kruskal-Wallis test for nonparametric valuables. Categorical variables were characterized by numbers with percentages and were compared using the Chi-square test. Univariate and multivariate logistic regression analysis helped to identify factors associated with disease severity. The variance inflation factor (VIF) detected multicollinearity among the independent variables. Correlations between miRNA expression levels and other variables were analyzed using the Pearson or Spearman correlation test as appropriate. The receiver operating characteristic (ROC) curve analysis was applied to evaluate the predictability of miRNAs. A *p*-value of less than 0.05 was considered statistically significant. All statistical analysis was done with SPSS Version 22 (SPSS, Chicago, IL). Figures were drawn using GraphPad Prism 8 (GraphPad Software Inc., California, USA).

## Results

### Patient characteristics

A total of 19 patients, 11 non-severe (with or without warning signs) and 8 severe, were selected for microRNA transcriptomic analysis in the discovery phase. Characteristics of the two groups are presented in Table 1. Compared to the non-severe group, patients with severe dengue had significantly higher AST and ALT levels but a lower DENV RT-PCR positive rate. A second sample of 135 patients (112 non-severe and 23 severe) were chosen for RT-qPCR in the validation phase. Compared to the non-severe group, patients with severe dengue had a significantly higher body temperature, diastolic blood pressure, respiratory rate, and AST level. In addition, severe dengue patients had a lower anti-DENV IgM positive rate, as well as lower rates of dengue warning signs. Other baseline characteristics were comparable between groups.

**Table 1:** Patient characteristics at enrollment.

Clinical Characteristics	Discovery Set (Nanostring)			Validation set (RT-qPCR)			Missing values, n (%)
	Non-severe (N = 11)	Severe (N = 8)	*p*-value	Non-severe (N = 112)	Severe (N = 23)	*p*-value	
**Demographic data**							
Age; years (median, IQR)	25.0 (16.0, 35.0)	17.0 (14.8, 21.8)	0.185	19.5 (14.0, 29.3)	17.0 (12.0, 28.0)	0.223	2 (1.5)
Sex; male (N, %)	5 (45.5)	5 (62.5)	0.463	48 (43.6)	13 (56.5)	0.259	2 (1.5)
BMI; kg/m^2^ (mean, SD)	21.3 (4.2)	24.2 (6.4)	0.303	21.6 (5.8)	20.4 (4.5)	0.823	17 (12.6)
Smoking (N, %)	1 (12.5)	0 (0.0)	0.333	7 (6.3)	1 (4.3)	0.922	0 (0)
Duration of fever at the time of recruitment; days (median, IQR)	2.0 (1.0, 4.0)	4.0 (3.0, 5.0)	0.097	4.0 (2.0, 4.0)	3.0 (2.8, 4.0)	0.344	3 (2.2)
**Physical examination**							
Body temperature; °C (median, IQR)	38.0 (37.3, 38.7)	38.0 (37.0, 39.4)	0.967	37.5 (36.8, 38.3)	38.9 (37.7, 39.9)	0.001*	2 (1.5)
SBP; mmHg (median, IQR)	113.0 (107.0, 120.0)	121.0 (110.0, 130.0)	0.405	109.0 (100.0, 117.8)	110.0 (100.0, 130.0)	0.361	2 (1.5)
DBP; mmHg (median, IQR)	62.0 (60.0, 70.0)	75.0 (63.5, 90.0)	0.079	61.0 (60.0, 70.0)	70.0 (60.0, 80.0)	0.005*	2 (1.5)
Respiratory rate; bpm (median, IQR)	20.0 (20.0, 20.0)	20.0 (20.0, 27.0)	0.241	20.0 (20.0, 24.0)	22.0 (20.0, 26.0)	0.007*	2 (1.5)
**Laboratory finding**							
Hemoglobin; g/dL (median, IQR)	13.2 (12.1, 14.1)	14.5 (11.8, 15.6)	0.264	13.3 (12.3, 14.4)	13.7 (12.2, 15.4)	0.394	3 (2.2)
HCT; % (median, IQR)	40.6 (34.1, 42.0)	43.9 (34.7, 46.0)	0.248	40.4 (37.5, 42.9)	40.5 (35.1, 46.2)	0.857	3 (2.2)
WBC; cells/μL (median, IQR)	2.46 (1.7, 4.1)	3.2 (2.3, 6.1)	0.322	3.1 (2.3, 4.4)	3.0 (2.2, 3.8)	0.746	3 (2.2)
Platelets; cells/μL (median, IQR)	96.0 (69.0, 104.0)	66.5 (46.3, 132.5)	0.741	96.0 (60.5, 145.5)	69.0 (50.0, 103.0)	0.068	3 (2.2)
Creatinine; mg/dL (median, IQR)	0.8 (0.6, 0.9)	0.8 (0.7, 1.4)	0.602	0.8 (0.6, 0.9)	0.8 (0.7, 1.3)	0.483	51 (37.8)
Glomerular filtration rate: mL/min (mean, SD)	100.1 (24.7)	117.4 (45.0)	0.385	99.3 (22.7)	95.3 (45.5)	0.761	56 (41.5)
Albumin; g/dL (median, IQR)	3.8 (3.4, 4.7)	3.6 (3.0, 4.4)	0.431	4.0 (3.6, 4.0)	3.6 (3.4, 4.0)	0.262	81 (60.0)
Total bilirubin; g/dL (median, IQR)	0.5 (0.3, 0.7)	0.7 (0.5, 1.5)	0.150	0.5 (0.4, 0.6)	0.5 (0.4, 1.1)	0.696	89 (65.9)
Direct bilirubin; g/dL (median, IQR)	0.3 (0.2, 0.4)	0.3 (0.2, 0.7)	0.647	0.3 (0.2, 0.4)	0.3 (0.2, 0.4)	0.725	90 (66.7)
AST; U/L (median, IQR)	49.0 (20.5, 118.5)	600.0 (105.0, 1061.0)	0.009*	106.5 (48.0, 146.3)	142.0 (92.0, 324.5)	0.037*	72 (53.3)
ALT; U/L (median, IQR)	32.0 (11.0, 103)	182.0 (54.0, 545.0)	0.020*	54.0 (29.5, 93.5)	67.0 (35.5, 120.0)	0.324	73 (54.1)
DENV RT-PCR positive (N, %)	7 (70.0)	1 (12.5)	0.015*	31 (28.2)	2 (9.1)	0.059	0 (0)
DENV NS1 positive (N, %)	10 (90.9)	7 (87.5)	0.811	76 (67.9)	20 (87.0)	0.066	0 (0)
Anti-DENV IgM positive (N, %)	3 (27.3)	2 (25.0)	0.912	71 (63.4)	9 (39.1)	0.031*	0 (0)
Anti-DENV IgG positive (N, %)	5 (45.5)	3 (37.5)	0.729	72 (64.3)	12 (52.2)	0.275	0 (0)
**Serotype** (N, %)			0.153			0.777	
DENV-1 (N, %)	1 (9.1)	1 (12.5)		13 (11.6)	2 (8.7)		
DENV-2 (N, %)	1 (9.1)	1 (12.5)		8 (7.1)	1 (4.3)		
DENV-3 (N, %)	3 (27.3)	0 (0.0)		4 (3.6)	0 (0.0)		
DENV-4 (N, %)	3 (27.3)	0 (0.0)		5 (4.5)	0 (0.0)		
DENV-1,3 (N, %)	0 (0.0)	0 (0.0)		1 (0.9)	0 (0.0)		
DENV-1,2,3 (N, %)	0 (0.0)	0 (0.0)		1 (0.9)	0 (0.0)		
Unknown (N, %)	3 (27.3)	6 (75.0)		80 (71.4)	20 (87.0)		
**Dengue warning sign (N, %)**	6 (54.5)	6 (75.0)	0.361	40 (36.4%)	18 (78.3%)	<0.001	
Presence abdominal pain or tenderness (N, %)	3 (27.3)	2 (25.0)	0.912	17 (15.5)	7 (30.4)	0.089	2 (1.5)
Persistent vomiting (N, %)	2 (18.2)	5 (62.5)	0.048*	20 (18.2)	11 (47.8)	0.002*	2 (1.5)
Clinical fluid accumulation (N, %)	0 (0.0)	0 (0.0)	NA	0 (0.0)	0 (0.0)	NA	2 (1.5)
Mucosal bleed (N, %)	1 (9.1)	1 (12.5)	0.811	11 (10.0)	3 (13.0)	0.665	2 (1.5)
Lethargy or restlessness (N, %)	0 (0.0)	0 (0.0)	NA	1 (0.9)	2 (8.7)	0.022*	2 (1.5)
Liver enlargement > 2 cm (N, %)	1 (9.1)	1 (12.5)	0.811	2 (1.8)	3 (13.0)	0.010*	2 (1.5)
Laboratory finding of increasing HCT concurrent with rapid decrease in platelet count (N, %)	2 (20.0)	3 (37.5)	0.410	3 (3.9)	3 (15.8)	0.055	39 (28.9)

BMI: body mass index, SBP: systolic blood pressure, DBP: diastolic blood pressure, HCT: Hematocrit, WBC: white blood cell, AST: aspartate transaminase, ALT: alanine aminotransferase, DENV: dengue virus, RT-PCR: *Reverse transcription polymerase chain reaction*, NA: not available,

*: *p*-value<0.05, Continuous data were expressed as means ± standard deviation (SD) or median and interquartile range (IQR). Categorical variables were expressed as numbers (%)

### MicroRNA Expression profile in serum

**Fig 1:**
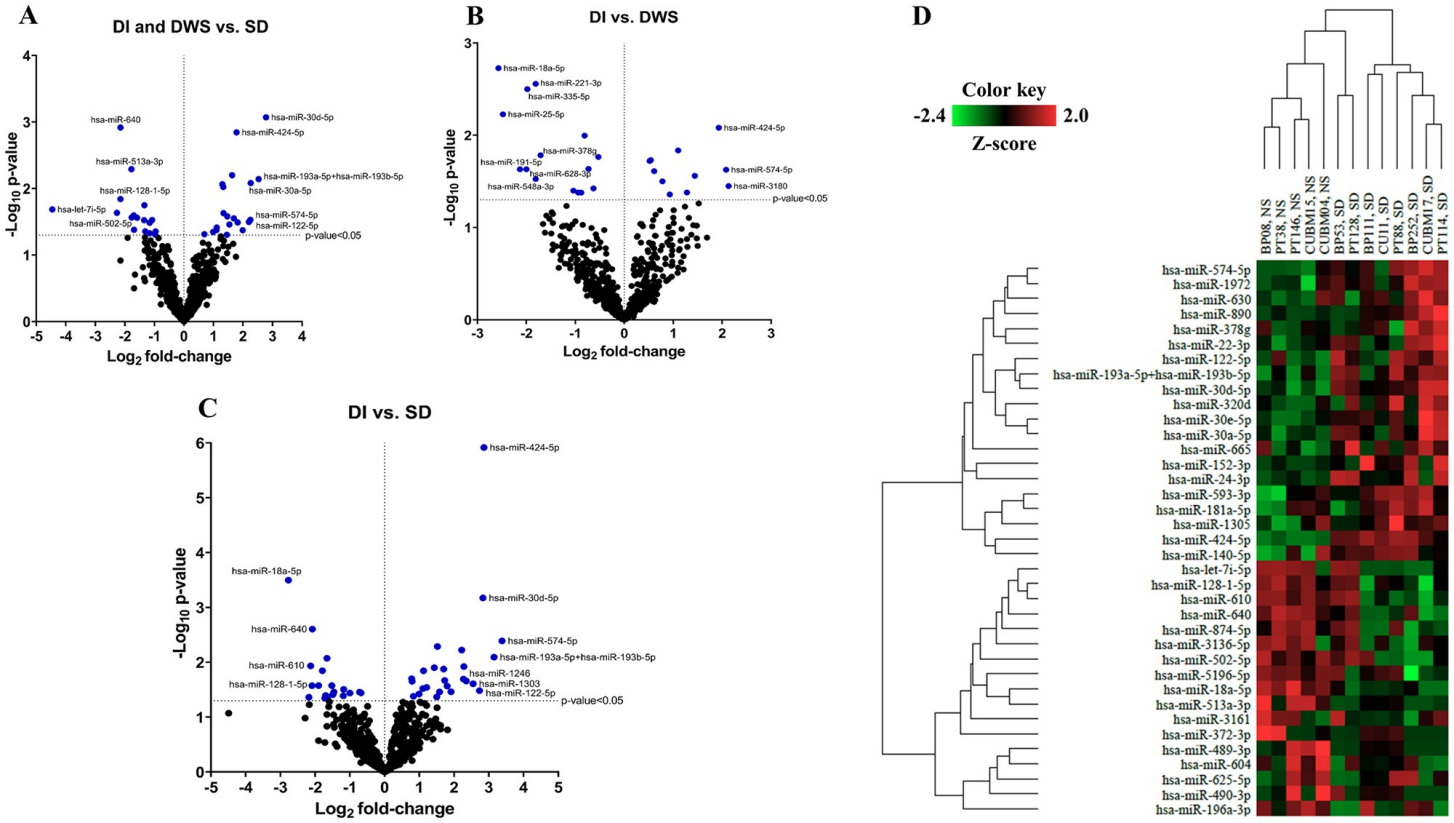
Circulating miRNA profiling of dengue-infected patients. A.Volcano plots showing changes in the expression levels of miRNAs between severe and non-severe groups. B.Volcano plots showing changes in the expression levels of miRNAs between DWS and DI groups. C.Volcano plots showing changes in the expression levels of miRNAs between SD and DI groups. D.The heatmap shows the 29 miRNAs dysregulated (Log_2_ fold change >1.5 and *p*-value<0.05) in the SD groups when compared with the DI group. DI, dengue without warning sign; DWS, dengue with a warning sign; SD, severe dengue.

Of 798 miRNAs determined by the NanoString Platform, 37 miRNAs had significant differential expression between severe and non-severe group (*p*-value of <0.05) (Fig 1A). To identify the miRNA signatures in the serum that are specifically associated with dengue disease progression, we also compared the microtranscriptome data obtained through NanoString among the three dengue groups representing the main clinical forms of the disease. The discovery set was classified into three subgroups including DI, DWS, and SD. We compared the DWS and SD microtranscriptome against the DI group. The result showed eight common dysregulated miRNAs in the DWS and SD groups compared to the DI group. The 36 and 18 miRNAs were uniquely dysregulated in SD and DWS groups, respectively (S3 Fig). Volcano plots were depicted to identify the miRNAs with the most significant fold differences and statistical significance between DWS and SD, compared to the DI group (Fig 1B and 1C). The heatmap in Fig 1D shows the 29 dysregulated miRNAs (Log2 fold change >1.5 and *p*-value<0.05) in the SD groups compared to the DI group. We found an unsupervised clustering of samples based on the severity of the disease, with the SD group clustered closer together and clearly separated from the DI group.

### Validation of NanoString analysis using RT-qPCR

**Fig 2:**
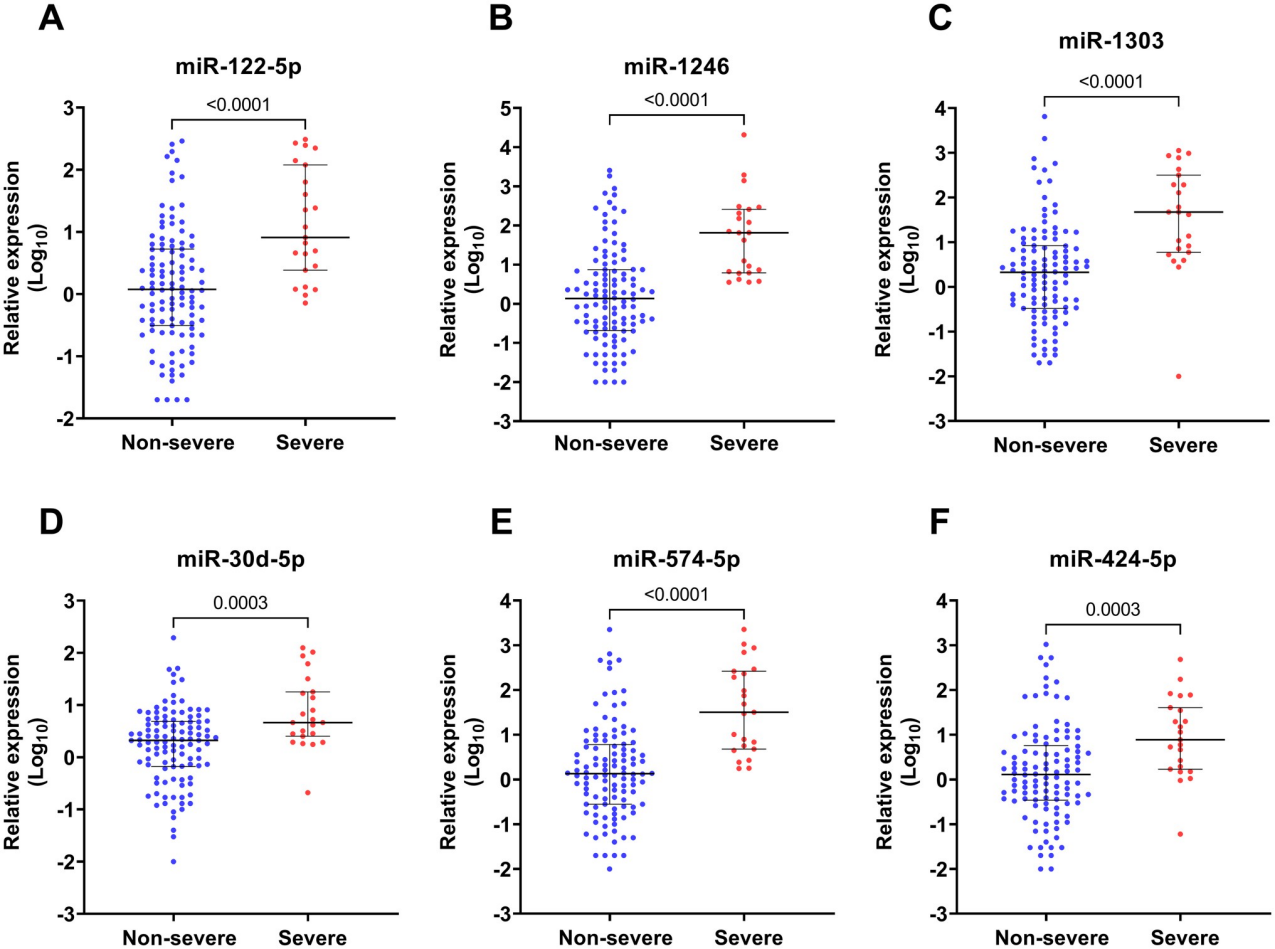
Relative expression of serum miRNAs in patients with severe and non-severe dengue.

To validate the results, six miRNAs (miR-122-5p, miR-574-5p, miR-424-5p, miR-1303, miR-30d-5p, and miR-1246) that had shown at least a two-fold upregulation on NanoString analysis were validated in the additional validation set, which consisted of 135 samples of patients with confirmed dengue. The RT-qPCR analysis confirmed the upregulation of all chosen miRNAs in severe dengue compared to the non-severe group. The relative expression of miRNAs levels of the subjects in each group are plotted in Fig 2. The results indicated that the circulating levels of six selected miRNAs were significantly higher in the severe dengue group compared to the non-severe group. The levels of the six selected circulating miRNAs were also significantly elevated in the serum of patients with SD compared to DWS and DI groups (S4 Fig). In addition, we found that the relative expression of miR122-5p, miR-1303, miR30d-5p, miR574-5p, and miR4245p was significantly higher in patients in the severe dengue group than in the non-severe, OFI, and healthy control group, indicated that these miRNAs could be potential specific biomarkers for severe dengue (S5 Fig).

### Relationship between circulating miRNAs levels and clinical parameters

Spearman correlations found a moderate to strong positive correlation between six miRNAs and several clinical parameters (S2 Table). Serum miR30d-5p and miR424-5p showed a positive correlation to serum creatinine and neutrophil levels. We also found miR122-5p, miR-1246, miR-1303, miR574-5p, and miR424-5p positively correlated with AST level and miR-574-5p positively correlated with ALT level. In contrast, we found several miRNAs negatively associated with platelets count, with miR-574-5p showing the strongest correlation, followed by miR-1246, miR-1303, and miR122-5p.

### Serum miRNAs as biomarkers of severe dengue

**Fig 3:**
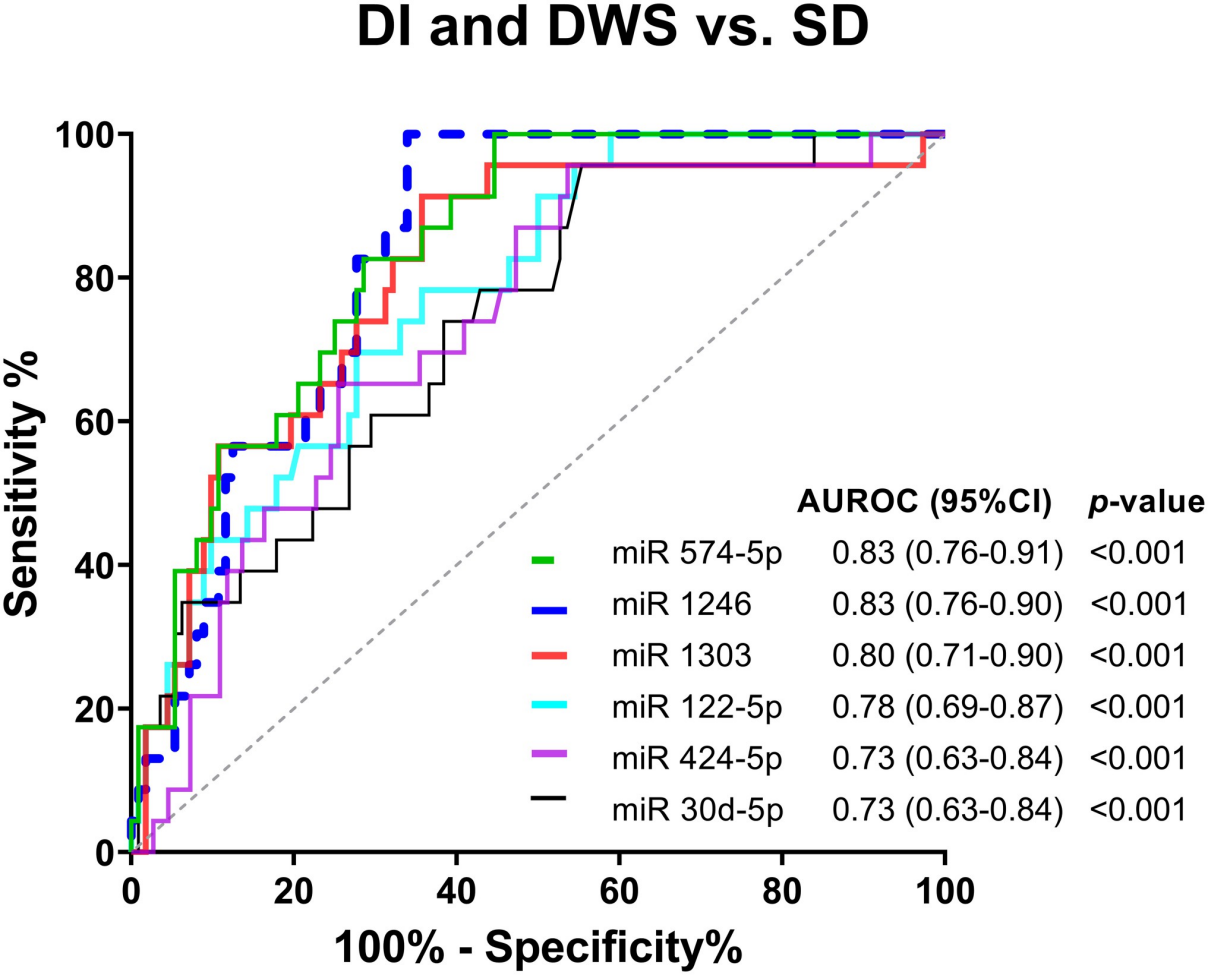
Receiver operating characteristic analysis of serum miRNAs for predicting severe dengue.

The ROC curves for six miRNAs were generated to calculate diagnostic accuracy. Six miRNAs could discriminate between the severe dengue and non-severe groups with an AUROC of 0.83 (95%CI;0.76–0.91, *p* <0.001) for miR-574-5p, 0.83 (95%CI;0.76–0.90, *p* <0.001) for miR-1246, 0.80 (95%CI;0.71–0.90, *p* <0.001) for miR-1303, 0.78 (95%CI;0.69–0.87, *p* <0.001) for miR-122-5p, 0.73 (95%CI;0.63–0.84, *p* <0.001) for miR-424-5p and 0.73 (95%CI;0.63–0.84, *p* = 0.001) for miR-30d-5p (Fig 3). In addition, we found that miRNAs are better predictors than other clinical biomarkers like persistent vomiting (AUC = 0.65, 95%CI;0.52–0.78, *p* = 0.026) and body temperature (AUC = 0.73, 95%CI;0.61–0.85, *p* = 0.001) as shown in S6 Fig.

We also compared the predictive accuracy of serum miRNAs on different disease days (day of defervescence was defined as day 0). The result showed that all six miRNAs distinguished severe dengue from non-severe in 1 to 2 days prior to the onset of severe complications which included shock from plasma leakage, acute liver failure, and acute kidney injury. However, there was no statistical significance when using these biomarkers on days 3 to 4 before the onset of severe dengue complications (Table 2).

**Table 2:** Receiver operator characteristics (ROC) curves comparing the predictive accuracy of serum miRNAs for predicting severe dengue on different disease days.

Defervescence day	miRNAs	AUC	95% CI		*p*-value
Day -4 to -3 (N = 19)	miR-122-5p	0.61	0.33	0.90	0.459
	miR-1246	0.69	0.42	0.95	0.229
	miR-1303	0.73	0.47	0.98	0.139
	miR-30d-5p	0.71	0.45	0.98	0.165
	miR-574-5p	0.73	0.47	0.98	0.139
	miR-424-5p	0.63	0.33	0.92	0.405
Day -2 to -1 (N = 63)	miR-122-5p	0.75	0.61	0.89	0.008*
	miR-1246	0.81	0.70	0.93	0.001*
	miR-1303	0.85	0.74	0.95	<0.001
	miR-30d-5p	0.76	0.64	0.89	0.005*
	miR-574-5p	0.84	0.73	0.95	<0.001
	miR-424-5p	0.76	0.64	0.89	0.005*

Day 0 (defervescence) was defined as the day on which the patient’s temperature fell and stayed below 37.5°C.

In addition, the AUROC curves of the combined six miRNAs were analyzed to understand if combined miRNAs detection could provide increased diagnostic power. Results showed that the combination of miRNAs expression did not improve the predictive performance. Based on the ROC, the optimal cut-off values for six miRNAs levels and their sensitivity, specificity, positive predictive value (PPV), and negative predictive value (NPV) in predicting severe dengue are shown in Table 3. Both miR-1246 and miR-574-5p displayed high sensitivity (100%) with high NPV (100%). We also performed subgroup analysis for children and adults (<15 versus ≥15 years of age). The result indicated that both sensitivity and NPV of miR-1246 and miR-574-5p were not different between the two age groups. These results confirm that serum miRNAs can identify severe dengue and non-severe with very high sensitivity and very high NPV for multiple age groups.

**Table 3:** Performance of serum miRNAs in the prediction of severe dengue.

miRNAs	Cut-off values	All age group				Children				Adults			
		Sen	Spec	PPV	NPV	Sen	Spec	PPV	NPV	Sen	Spec	PPV	NPV
miR-122-5p	2.4	78	63	31	93	73	48	35	82	83	68	28	96
miR-1246	3.5	100	66	38	100	100	55	46	100	100	69	32	100
miR-1303	3.8	87	64	33	96	82	69	50	91	92	62	26	98
miR-30d5p	1.8	91	46	26	96	100	52	44	100	83	42	18	94
miR-5745p	1.7	100	55	32	100	100	62	50	100	100	52	24	100
miR-424-5p	0.9	96	45	27	98	91	52	42	94	100	42	21	100

Sen: Sensitivity, Spec: Specificity, PPV: Positive predictive value, NPV: Negative predictive value

### Univariable and multivariable regression analysis

Serum miRNAs and other variables that might influence the prognosis of severe dengue were entered into the univariate analysis. The data revealed that prognostic factors of severe dengue were body temperature, neutrophils, anti-DENV IgM positive, persistent vomiting, liver enlargement, laboratory finding of increasing HCT concurrent with a rapid decrease in platelet count, miR-122-5p, miR-1246, miR-1303, miR-574-5p, miR-30d-5p, and miR-424-5p. Since there were correlations among the six miRNAs, only miR-574-5p was selected for the multivariate analysis together with the other variables shown significant in the univariate analysis. Multivariate regression results indicated that body temperature (unit change of one degree Celsius), persistent vomiting (vomiting three times or more per day), and miR-574-5p were independent predictors of severe dengue (Table 4).

**Table 4:** Variables associated with severe dengue.

Variables	Univariate analysis			Multivariate analysis		
	OR	95%CI	*p*-value	OR	95%CI	*p*-value
Age	0.99	0.96–1.03	0.722			
Gender	1.68	0.68–4.16	0.262			
Body mass index	0.99	0.90–1.08	0.821			
Duration of fever at the time of recruitment; days	0.85	0.63–1.14	0.282			
Body temperature	2.03	1.37–3.00	<0.001	2.38	1.31–4.33	0.005*
Hematocrit	1.00	0.92–1.08	0.917			
White blood cell	0.93	0.72–1.19	0.559			
Platelets	0.99	0.98–1.00	0.065			
Neutrophils	1.04	1.01–1.07	0.011*	1.01	0.97–1.06	0.541
Aspartate transaminase	1.00	1.00–1.00	0.054			
Alanine aminotransferase	1.00	1.00–1.01	0.484			
DENV RT-PCR positive	0.38	0.10–1.35	0.133			
DENV NS1 positive	3.16	0.88–11.32	0.077			
Anti-DENV IgM positive	0.37	0.15–0.93	0.035*	0.76	0.21–2.73	0.671
Anti-DENV IgG positive	0.61	0.25–1.50	0.278			
Presence abdominal pain or tenderness	2.39	0.86–6.69	0.096			
Persistent vomiting	4.12	1.59–10.68	0.003*	9.20	2.15–39.42	0.003*
Mucosal bleed	1.35	0.35–5.28	0.666			
Lethargy or restlessness	10.38	0.90–119.76	0.061			
Liver enlargement	8.10	1.27–51.60	0.027*	4.70	0.39–56.87	0.224
Laboratory finding of increasing HCT concurrent with rapid decrease in platelet count	5.45	1.03–28.95	0.047*	3.19	0.36–28.66	0.300
miR-122-5p (Log10)	2.94	1.74–4.97	<0.001			
miR-1246 (Log10)	2.61	1.70–4.01	<0.001			
miR-1303 (Log10)	2.46	1.59–3.82	<0.001			
miR-574-5p (Log10)	3.06	1.90–4.95	<0.001	3.30	1.81–6.04	<0.001
miR-30d-5p (Log10)	3.70	1.75–7.81	0.001*			
miR-424-5p (Log10)	2.04	1.29–3.21	0.002*			

DENV: dengue virus, RT-PCR: Reverse transcription polymerase chain reaction,

*: *p*-value<0.05

### Association of serum miRNAs with dengue clinical outcomes

We further explored the associations of each miRNA with other clinical outcomes (see S3 Table), including plasma leakage (defined as a rise in HCT ≥20%), thrombocytopenia (defined as platelet count <20,000/cu.mm), and mild mucosal bleeding. The result indicated that miR-122-5p (*p* = 0.018), miR-1246 (*p* = 0.003), miR-1303 (*p* = 0.007), and miR-574-5p (*p* = 0.006) were associated with plasma leakage. In addition, the level of miR-1246 (*p* = 0.049) and miR-1303 (*p* = 0.045) was related to mild mucosal bleeding. We did not find any association between serum miRNAs and thrombocytopenia in patients with dengue fever.

## Discussion

Dengue is a potentially life-threatening viral infection that affects approximately 100 million people per year worldwide [29]. Identifying early prognostic markers of severe complications may improve case management and reduce dengue-related mortalities [2] This study aimed to identify miRNA expression profiles in patients with severe dengue, which might be used as biomarkers for predicting severe dengue and validating their applications. In the discovery cohort, we identified 37 miRNAs differentially expressed between the two study groups. The top six candidates miRNAs selected were miR-122-5p, miR-574-5p, miR-424-5p, miR-1303, miR-30d-5p, and miR-1246. We validated the miRNAs in a multi-center prospective cohort in Thailand. The RT-qPCR analysis confirmed that the levels of six miRNAs were significantly higher in patients with severe dengue compared to the non-severe group. These miRNAs concentrations were also positively correlated with clinical parameters such as neutrophil, creatinine, and AST level but negatively correlated with platelet counts. Based on the ROC curve, it was shown that six circulating miRNAs could discriminate severe dengue from the non-severe samples. Both miR-1246 and miR-574-5p showed the best diagnostic performance with an AUC of 0.83 and high sensitivity (100%) with NPV (100%). We found that the combination of miRNAs expression did not improve the predictive performance. In addition, our results showed that both miRNAs performed equally in DENV-infected children and adults, indicating that it is not affected by age-dependent variations in immune responses. In predicting severe dengue (1–2 days before defervescence), the miR-1303 or miR-574-5p alone exhibited good sensitivity and specificity with an AUC of 0.85 and 0.84, respectively, which is considered as an excellent predictive performance. According to the study design, serum samples of all patients were collected for the miRNAs expression analysis at the time of admission, implying that the serum miRNAs level alone might be used to predict severe dengue upon hospital admission, especially when using these biomarkers on days 1 to 2 before the onset of severe dengue complications.

Downregulated miRNAs can also be biomarkers, as described in many studies [30–32]. We also performed RT-qPCR validation on the top two down-regulated miRNA (miR-18a-5p and miR-640) identified by Nanostring analysis. However, the result showed no differences in the two miRNAs between the non-severe and severe-dengue groups (data do not show).

Previous work has noted that thrombocytopenia and elevated AST/ALT in the first 72 hours post fever onset are early predictors of severe dengue [33,34]. However, in our cohort, these variables could not distinguish deterioration to severe dengue in the 3 to 4 days and 1 to 2 days before the onset of severe dengue (data do not show).

Previous studies have reported differential expression of miRNAs in dengue patients and infected cultured cells [14–17]. However, the role of miRNAs in severe dengue is still not completely understood. In a recent study, small RNA sequencing data obtained from the plasma of 39 adult dengue patients revealed that some of the miRNAs were differentially expressed during the different stages of dengue infection. Among these miRNAs, miR-320a-5p, miR-486-5p, and miR-122-5p could distinguish between patients with uncomplicated dengue infection and severe dengue cases with an AUC of 0.81, 0.73, and 0.79, respectively [35]. The present study is more comprehensive because it encompasses a larger prospective validation cohort with a broader age range of patients.

This is the first study that described the association of miR-574-5p with severe dengue. In lung cancer, Li et al. showed that miRNA-574-5p was pivotal for Toll-like receptor 9 (TLR9) signaling, enhanced tumor progression via downregulating Forkhead box N3 (FOXN3) [36]. TLR are reported to play a major role in regulating inflammatory response against infectious viruses [37,38]. A previous study reported that DENV infection induces the release of mitochondrial DNA (mtDNA) into the cytosol and activates TLR9 signaling pathways, leading to the production of interferons (IFNs) [39]. Taken together, these findings suggest that the regulation of TLR9 signaling by miRNA-574-5p and FOXN3 might have an impact on the development of severe dengue and deserves further study to clarify this relationship.

MiR-1246 has been found to be upregulated in DENV2-infected PBMC compared to uninfected control [14]. Its role in severe dengue pathogenesis remains unclear. MiR-1246 could promote metastasis and invasion in lung cancer cells by targeting glycogen synthase kinase-3β (GSK-3β)-mediated Wnt/β-catenin pathway [40]. Notably, it was found that DENV-2 inhibits GSK-3 activity to induce expression of MHC Class-1-related chain (MIC) A and MIC-B, and IL-12 production in monocyte-derived dendritic cells [41]. Moreover, a recent study demonstrated that GSK-3β participates at the late stages of the DENV replication cycle, where viral activation may promote apoptosis and the release of viral particles.

MiR-1303 was also upregulated in this study. Toll-like receptor 4 (TLR4) is also one of the targets of miR-1303 [42]. Modhiran et al. demonstrated that DENV NS-1 protein activates mouse macrophages and human PBMC cells via TLR4 and disrupts endothelial cell monolayer integrity [43]. A recent study also reported that DENV NS-1 activates platelets via TLR4, which leads to thrombocytopenia and hemorrhage [44].

Consistent with previous research, this study also confirms that miR-122-5p was upregulated in severe dengue. The miR-122-5p is a liver-specific miRNA and plays a pivotal role in lipid metabolism, tumor suppression, and liver homeostasis [45]. Tambyah et al. and Saini et al. reported that miR-122-5p was significantly upregulated in the blood [16] and plasma [35] of the severe dengue patients and could be used to differentiate between different stages of dengue infection under the current WHO classification criteria (AUC = 0.79). Further research is needed to determine the role of this miRNA in the pathogenesis of severe dengue.

Regarding miR-424-5p, suppressor of cytokine signaling 2 (SOCS2) [46] was a direct target of this miRNA. SOCS proteins are negative feedback regulators of the Janus kinase/signal transducer and activator of transcription (JAK/STAT) pathway [47]. MiR-30d-5p can target the suppressor of cytokine signaling 3 (SOCS3) and affect the JAK/ STAT3 signaling pathway.[48] Previous data showed that DENV infection induced high expression of SOCS3 in macrophages, and its changes were associated with evasion of the antiviral innate immune response [49].

Thrombocytopenia is a common feature observed in non-severe and severe dengue and is associated with the disease severity [50,51]. Several factors were identified as risk factors associated with the development of thrombocytopenia in dengue [52]. MiRNAs have been described to have essential roles in primary immune thrombocytopenia (ITP) in several studies [53,54]. To our knowledge, there is no study on the role of miRNAs and thrombocytopenia in dengue. In this study, we report, for the first time, that there was no association between serum miRNAs and thrombocytopenia in dengue patients.

To our knowledge, this study contained the largest sample size with the broadest age range of patients compared to previously published studies to investigate the association between the levels of circulating miRNAs and dengue infection outcomes. This study also had serum samples from patients infected with all four DENV serotypes; however, serotype-specific differences were not analyzed because of the small sample sizes. In addition, the high dengue IgG positivity rate indicates a secondary infection in this population. This study can be a good reference in future studies on miRNAs.

This study had some limitations. Firstly, we analyzed the miRNAs at only one-time point on the first day of enrollment. We did not measure levels when the patient’s condition changed or after the intervention had been given. Measurements at different time points may help us to better understand the changes of these miRNAs during the development of dengue illness. Secondly, we have a low number of cases in key severe dengue subgroups such as acute kidney injury or acute liver injury. Further studies are needed to determine whether our results are valid for different types of dengue severity. Thirdly, it is well known that the sensitivities of the rapid test for anti-DENV IgM and IgG antibodies are poor. The serological status should be confirmed by ELISA for accuracy. However, this study reflects actual clinical settings. In addition, our study included only Thai patients with dengue infection; most patients had unknown DENV serotypes. Finally, the sample size is quite small when we perform AUC subgroup analysis, especially on day 3 or 4 prior to the development of severe symptoms which might affect our results. The differential expression of the miRNAs should be studied in an extended cohort with diverse populations to validate the miRNAs identified in this study.

In conclusion, our study indicated that circulating miRNAs, especially miR-574-5p and miR-1246, might be a promising diagnostic and prognostic biomarker for severe dengue upon hospital admission, especially when using these biomarkers on days 1 to 2 before the onset of severe dengue complications. This miRNA analysis should be further validated in larger prospective cohorts with the biological functions of these miRNAs requiring further investigation.

## Supporting information

S1 Table Primer sequences for miRNAs used in the study. (DOCX)

S2 Table Relationship between serum miRNAs levels and clinical parameters. (DOCX)

S3 Table Association between miRNAs with plasma leakage, thrombocytopenia, and mild mucosal bleeding (represented by *p*-value). (DOCX)

S1 Fig RT-qPCR standardization. (DOCX)

S2 Fig The RT-qPCR cycle threshold (CT) values for miR-16-5p in the serum samples. (DOCX)

S3 Fig The Venn diagram represents the common and differentially expressed mRNAs in the DWS and SD groups when compared with the DI group. (DOCX)

S4 Fig Relative expression of serum miRNAs in patients with dengue infection, dengue with a warning sign, and severe dengue. (DOCX)

S5 Fig Relative expression of serum miRNAs in healthy controls and other febrile illness patients. (DOCX)

S6 Fig Receiver operating characteristic analysis of clinical markers for predicting severe dengue. (DOCX)
