# Supplementary material for: Discovery and validation of circulating miRNAs for the clinical prognosis of severe dengue
Source: PLoS Negl Trop Dis. 2022 Oct 17;16(10):e0010836. doi: 10.1371/journal.pntd.0010836 (PMC9576100; doi:10.1371/journal.pntd.0010836)
Supplement: S1 Table — (DOCX) [file pntd.0010836.s001.docx]

**S1 Table. Primer sequences for miRNAs used in the study.**

| **No** | **Primer name** | **Primer seq (5'-3')** |
| --- | --- | --- |
| 1 | hsa-miR-122-5p | GAGTGTGACAATGGTGTTTG |
| 2 | hsa-miR-1246 | GAATGGATTTTTGGAGCAGG |
| 3 | hsa-miR-30d-5p | TAAACATCCCCGACTGGAAG |
| 4 | hsa-miR-424-5p | CAGCAGCAATTCATGTTTTG |
| 5 | hsa-miR-512-5p | CAGCCTTGAGGGCACTTTC |
| 6 | hsa-miR-574-5p | TGAGTGTGTGTGTGTGAGTG |
| 7 | hsa-miR-1303 | TAGAGACGGGGTCTTGCTC |
| 8 | hsa-miR-18a-5p | TAAGGTGCATCTAGTGCAGATAG |
| 9 | hsa-miR-610 | AGCTAAATGTGTGCTGGGA |
| 10 | hsa-miR-640 | TGATCCAGGAACCTGCCT |
| 11 | hsa-miR-16-5p | CAGCACGTAAATATTGGCG |
| 12 | miRNA-rev | GCAGGGTCCGAGGTATTC |
| 13 | SL-poly(A) | GTCGTATCCAGTGCAGGGTCCGAGGTATTCGCACTGGATACGACAAAAAAAAAAAAAAAAAAVN |
