# Supplementary material for: Discovery and validation of circulating miRNAs for the clinical prognosis of severe dengue
Source: PLoS Negl Trop Dis. 2022 Oct 17;16(10):e0010836. doi: 10.1371/journal.pntd.0010836 (PMC9576100; doi:10.1371/journal.pntd.0010836)
Supplement: S2 Table — (DOCX) [file pntd.0010836.s002.docx]

**S2 Table. Relationship between serum miRNAs levels and clinical parameters**

| **Parameters** | **miR122-5p** | | **miR-1246** | | **miR-1303** | | **miR30d-5p** | | **miR574-5p** | | **miR424-5p** | |
| --- | --- | --- | --- | --- | --- | --- | --- | --- | --- | --- | --- | --- |
|  | **r** | **p** | **r** | **p** | **r** | **p** | **r** | **p** | **r** | **p** | **r** | **p** |
| miR122-5p |  |  | 0.899 | <0.001* | 0.789 | <0.001* | 0.678 | <0.001* | 0.854 | <0.001* | 0.833 | <0.001* |
| miR-1246 | 0.899 | <0.001* |  |  | 0.810 | <0.001* | 0.618 | <0.001* | 0.911 | <0.001* | 0.843 | <0.001* |
| miR-1303 | 0.789 | <0.001* | 0.810 | <0.001* |  |  | 0.632 | <0.001* | 0.877 | <0.001* | 0.761 | <0.001* |
| miR30d-5p | 0.678 | <0.001* | 0.618 | <0.001* | 0.632 | <0.001* |  |  | 0.698 | <0.001* | 0.750 | <0.001* |
| miR574-5p | 0.854 | <0.001* | 0.911 | <0.001* | 0.877 | <0.001* | 0.698 | <0.001* |  |  | 0.839 | <0.001* |
| miR424-5p | 0.833 | <0.001* | 0.843 | <0.001* | 0.761 | <0.001* | 0.750 | <0.001* | 0.839 | <0.001* |  |  |
| Hemoglobin | 0.118 | 0.178 | 0.166 | 0.058 | 0.044 | 0.618 | -0.037 | 0.670 | 0.118 | 0.176 | 0.162 | 0.066 |
| HCT | 0.087 | 0.320 | 0.141 | 0.107 | 0.012 | 0.896 | -0.096 | 0.272 | 0.075 | 0.394 | 0.142 | 0.106 |
| WBC | 0.069 | 0.429 | -0.039 | 0.655 | 0.054 | 0.536 | 0.153 | 0.081 | -0.014 | 0.877 | -0.063 | 0.480 |
| Platelets | -0.180 | 0.039* | -0.303 | <0.001* | -0.201 | 0.021 | 0.081 | 0.353 | -0.340 | <0.001* | -0.161 | 0.067 |
| Neutrophil | 0.150 | 0.085 | 0.107 | 0.224 | 0.106 | 0.224 | 0.318 | <0.001* | 0.117 | 0.181 | 0.227 | 0.009 |
| Creatinine | 0.159 | 0.150 | 0.149 | 0.175 | 0.131 | 0.236 | 0.216 | 0.048 | 0.208 | 0.058 | 0.248 | 0.025 |
| Glomerular filtration rate | -0.058 | 0.612 | -0.067 | 0.558 | -0.017 | 0.884 | -0.008 | 0.946 | -0.057 | 0.619 | -0.124 | 0.278 |
| Albumin | -0.104 | 0.456 | -0.102 | 0.461 | -0.227 | 0.099 | -0.099 | 0.477 | -0.156 | 0.261 | -0.011 | 0.937 |
| Total bilirubin | 0.207 | 0.168 | 0.239 | 0.110 | 0.171 | 0.256 | 0.118 | 0.436 | 0.197 | 0.189 | 0.120 | 0.427 |
| Direct bilirubin | 0.243 | 0.107 | 0.248 | 0.100 | 0.185 | 0.225 | 0.098 | 0.523 | 0.226 | 0.136 | 0.168 | 0.271 |
| AST | 0.383 | 0.002* | 0.420 | 0.001* | 0.344 | 0.006 | 0.128 | 0.316 | 0.470 | <0.001* | 0.250 | 0.048 |
| ALT | 0.230 | 0.072 | 0.218 | 0.089 | 0.165 | 0.199 | 0.016 | 0.900 | 0.273 | 0.032 | 0.085 | 0.510 |

HCT: Hematocrit, WBC: white blood cell, AST: aspartate transaminase, ALT: alanine aminotransferase, *: *p*-value<0.05,
