# Supplementary material for: Discovery and validation of circulating miRNAs for the clinical prognosis of severe dengue
Source: PLoS Negl Trop Dis. 2022 Oct 17;16(10):e0010836. doi: 10.1371/journal.pntd.0010836 (PMC9576100; doi:10.1371/journal.pntd.0010836)
Supplement: S3 Table — (DOCX) [file pntd.0010836.s003.docx]

**S3 Table. Association between miRNAs with plasma leakage, thrombocytopenia, and mild mucosal bleeding (represented by *p*-value).**

| **miRNAs** | **Plasma leakage** | **Thrombocytopenia** | **Mild mucosal bleeding** |
| --- | --- | --- | --- |
| miR122-5p | 0·018* | 0·656 | 0·163 |
| miR-1246 | 0·003* | 0·455 | 0·049* |
| miR-1303 | 0·007* | 0·773 | 0·045* |
| miR30d-5p | 0·132 | 0·479 | 0·053 |
| miR574-5p | 0·006* | 0·325 | 0·112 |
| miR424-5p | 0·196 | 0·958 | 0·144 |
