# Supplementary material for: Discovery and validation of circulating miRNAs for the clinical prognosis of severe dengue
Source: PLoS Negl Trop Dis. 2022 Oct 17;16(10):e0010836. doi: 10.1371/journal.pntd.0010836 (PMC9576100; doi:10.1371/journal.pntd.0010836)
Supplement: S1 Fig — (DOCX) [file pntd.0010836.s004.docx]

**S1 Fig. RT-qPCR standardization**

**
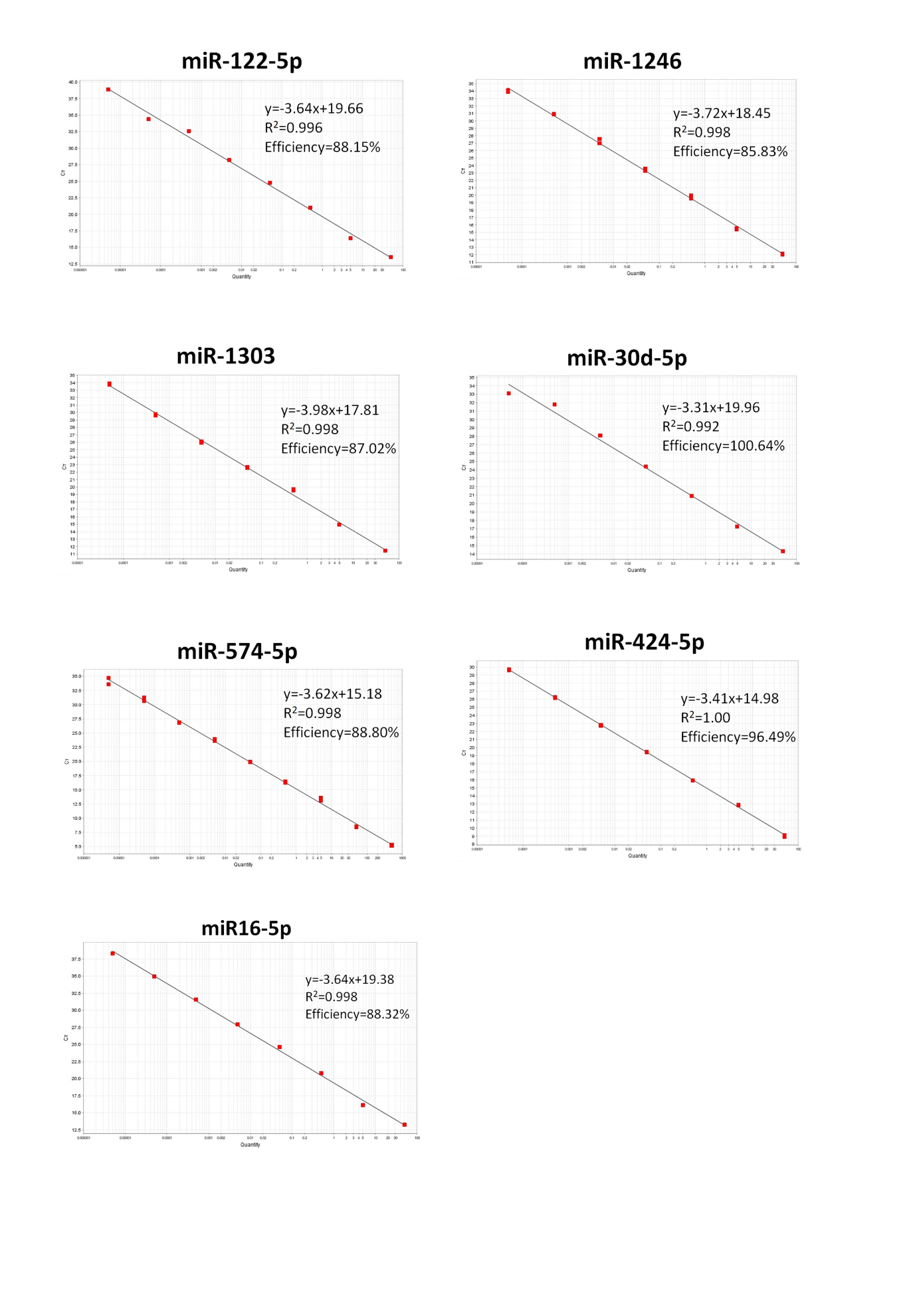
**Ten-fold serial dilution of synthetic miRNA was used to generate the standard curves. Linearity was confirmed within the concentration ranging from 50-0.000005 fmol. All tested assays had good linearity (R2 > 0.99) and acceptable PCR efficiency (slope was between −3.31 and −3.98, corresponding to PCR efficiencies between 85.83 and 100.64%)
