# Supplementary material for: Discovery and validation of circulating miRNAs for the clinical prognosis of severe dengue
Source: PLoS Negl Trop Dis. 2022 Oct 17;16(10):e0010836. doi: 10.1371/journal.pntd.0010836 (PMC9576100; doi:10.1371/journal.pntd.0010836)
Supplement: S3 Fig — (DOCX) [file pntd.0010836.s006.docx]

**
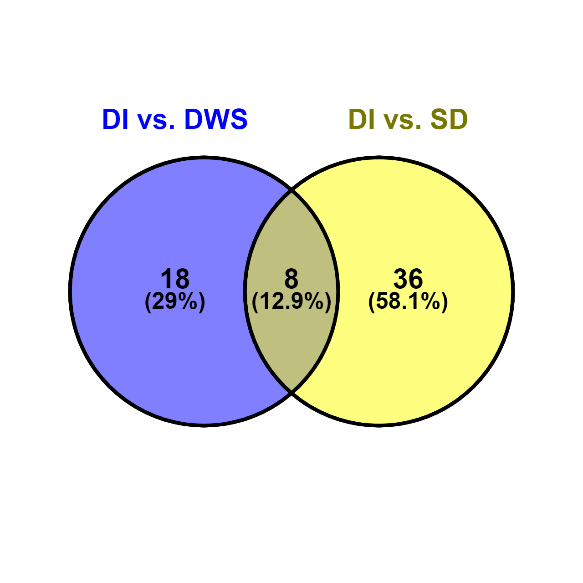
S3 Fig. The Venn diagram represents the common and differentially expressed mRNAs in the DWS and SD groups when compared with the DI group**

DI, dengue without warning sign; DWS, dengue with a warning sign; SD, severe dengue
