# Supplementary material for: Discovery and validation of circulating miRNAs for the clinical prognosis of severe dengue
Source: PLoS Negl Trop Dis. 2022 Oct 17;16(10):e0010836. doi: 10.1371/journal.pntd.0010836 (PMC9576100; doi:10.1371/journal.pntd.0010836)

**S4 Fig. Relative expression of serum miRNAs in patients with dengue infection, dengue with a warning sign, and severe dengue**

DI, dengue without warning sign; DWS, dengue with a warning sign; SD, severe dengue


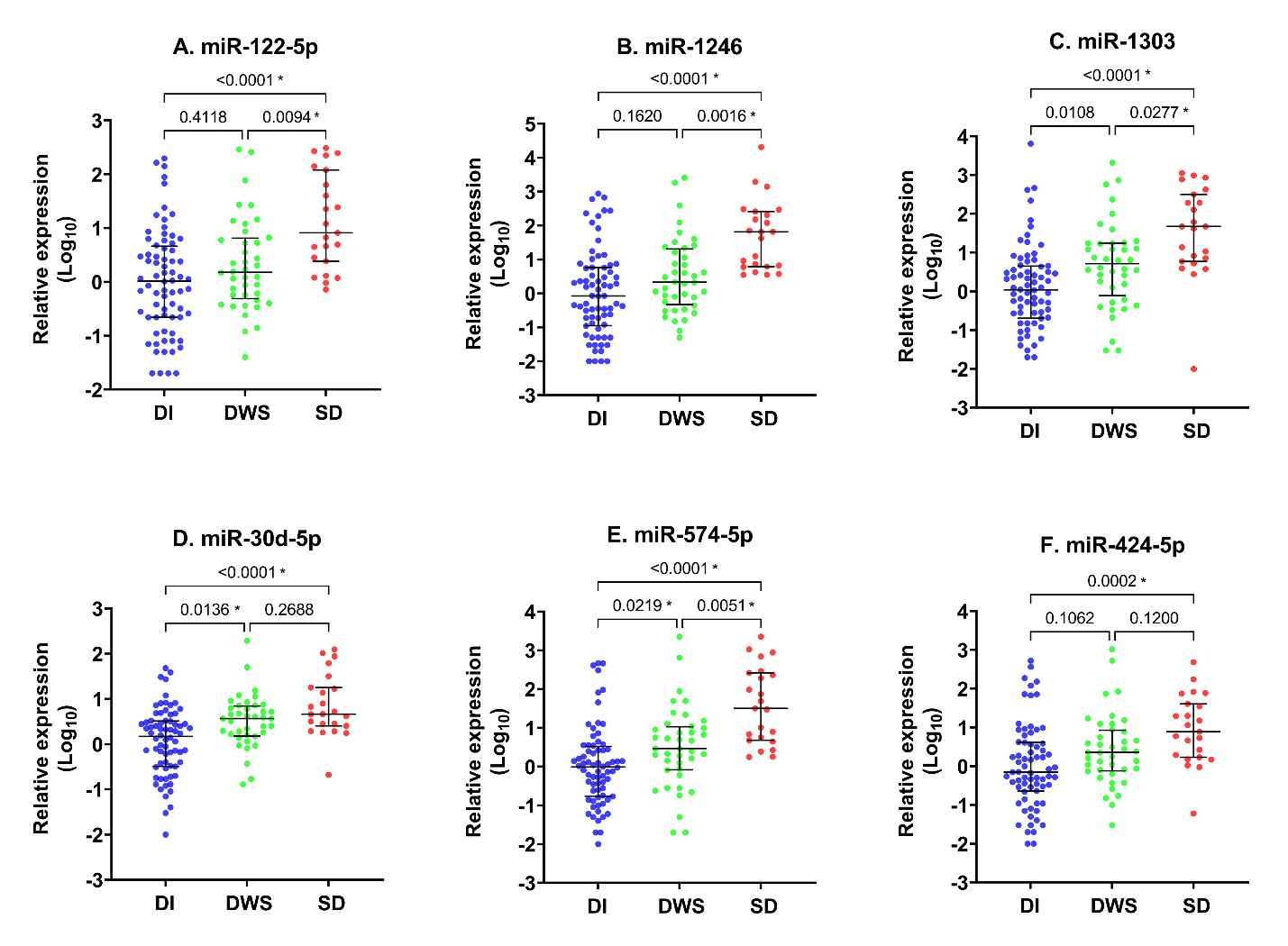

Supplement: S4 Fig — (DOCX) [file pntd.0010836.s007.docx]
