# Supplementary material for: Discovery and validation of circulating miRNAs for the clinical prognosis of severe dengue
Source: PLoS Negl Trop Dis. 2022 Oct 17;16(10):e0010836. doi: 10.1371/journal.pntd.0010836 (PMC9576100; doi:10.1371/journal.pntd.0010836)

**S5 Fig. Relative expression of serum miRNAs in healthy controls and other febrile illness patients.**

HC, healthy controls; OFI, other febrile illness patients; NS, non-severe dengue; SD, severe dengue


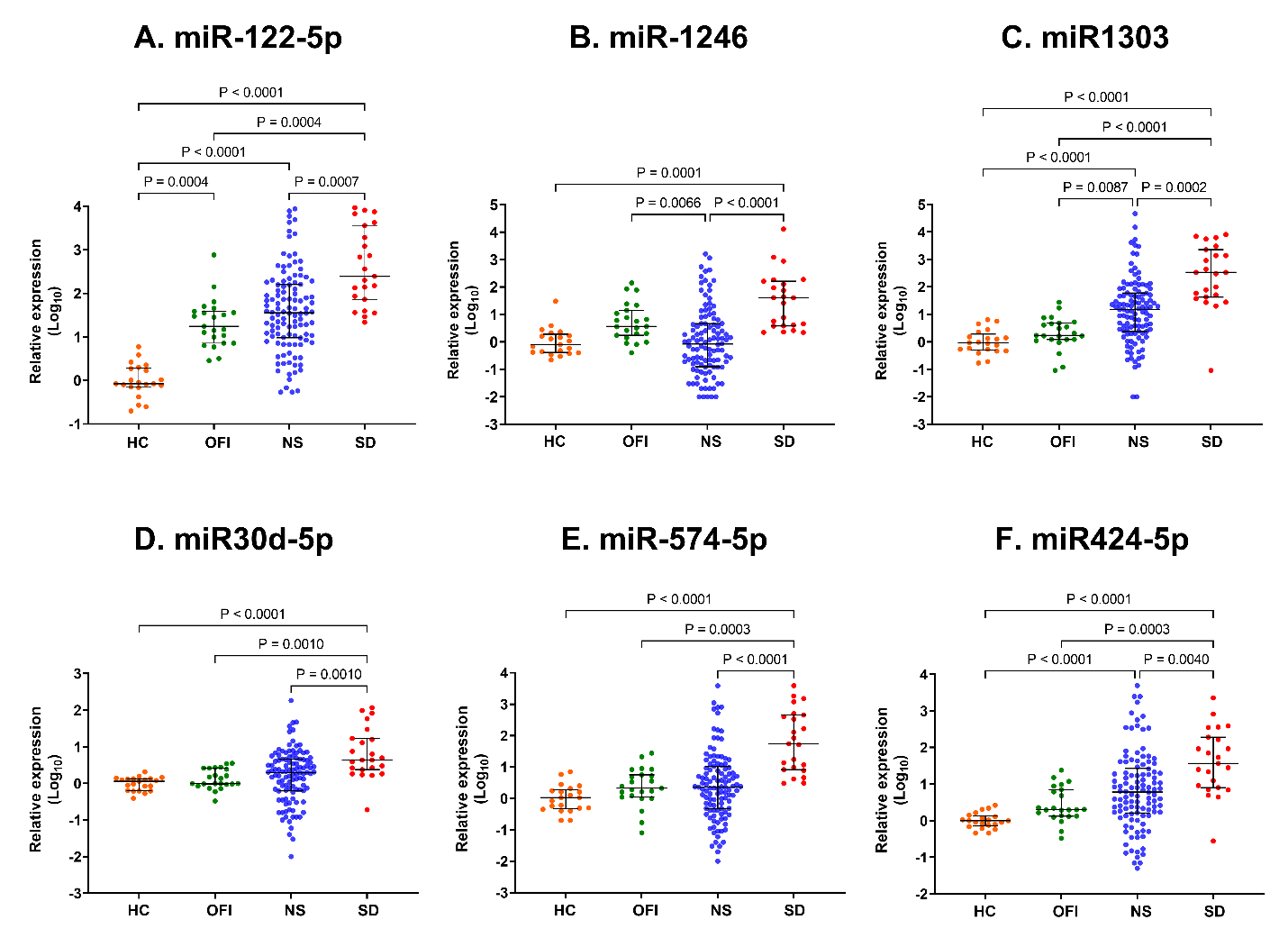

Supplement: S5 Fig — (DOCX) [file pntd.0010836.s008.docx]
