# Supplementary material for: Discovery and validation of circulating miRNAs for the clinical prognosis of severe dengue
Source: PLoS Negl Trop Dis. 2022 Oct 17;16(10):e0010836. doi: 10.1371/journal.pntd.0010836 (PMC9576100; doi:10.1371/journal.pntd.0010836)
Supplement: S6 Fig — (DOCX) [file pntd.0010836.s009.docx]

**
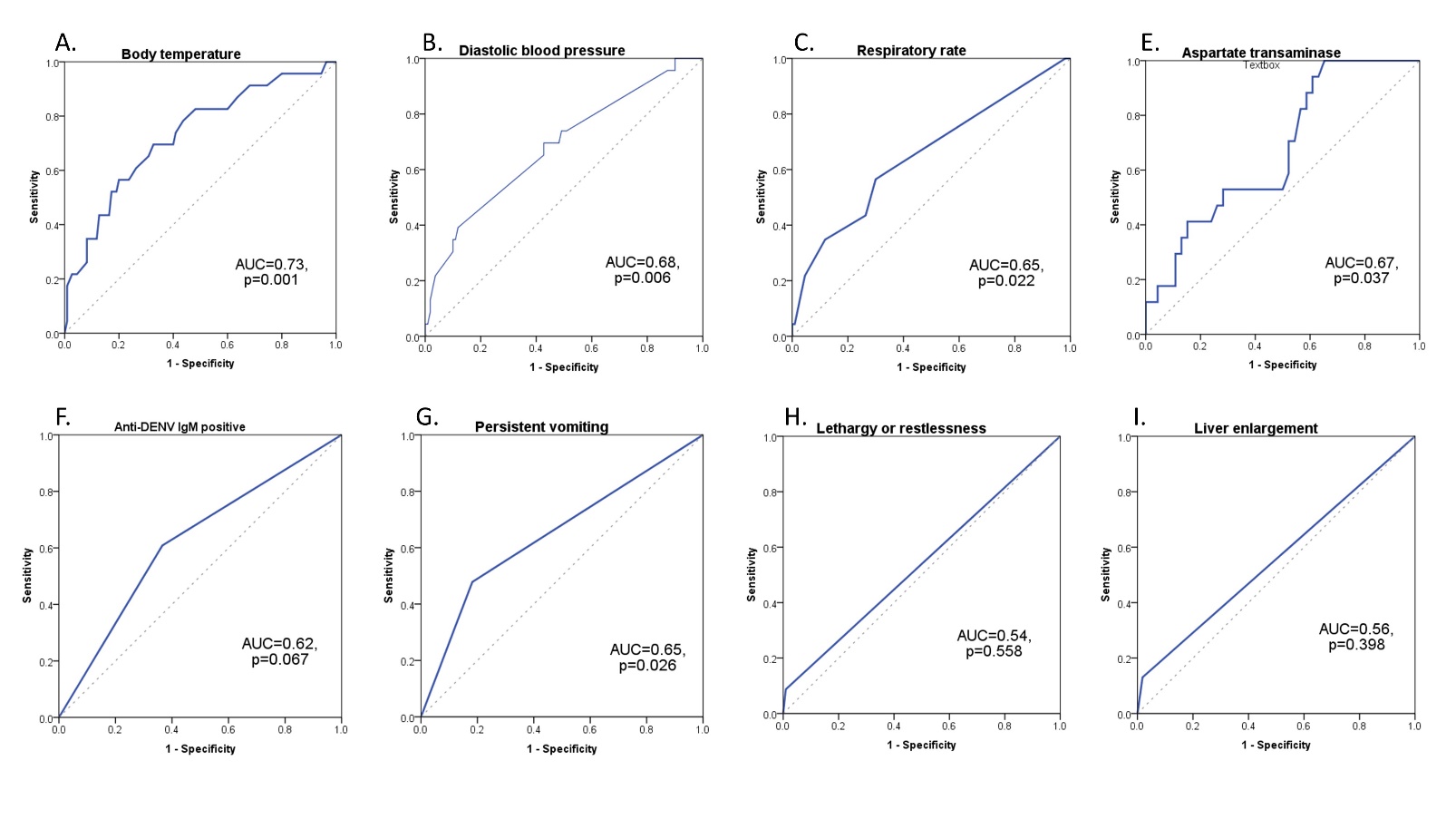
S6 Fig. Receiver operating characteristic analysis of clinical markers for predicting severe dengue**
